# Chromatin Modulation of Herpesvirus Lytic Gene Expression: Managing Nucleosome Density and Heterochromatic Histone Modifications

**DOI:** 10.1128/mBio.00098-16

**Published:** 2016-02-16

**Authors:** Thomas M. Kristie

**Affiliations:** Laboratory of Viral Diseases, National Institute of Allergy and Infectious Diseases, National Institutes of Health, Bethesda, Maryland, USA

## Abstract

Like their cellular hosts, herpesviruses are subject to the regulatory impacts of chromatin assembled on their genomes. Upon infection, these viruses are assembled into domains of chromatin with heterochromatic signatures that suppress viral gene expression or euchromatic characteristics that promote gene expression. The organization and modulation of these chromatin domains appear to be intimately linked to the coordinated expression of the different classes of viral genes and thus ultimately play an important role in the progression of productive infection or the establishment and maintenance of viral latency. A recent report from the Knipe laboratory (J. S. Lee, P. Raja, and D. M. Knipe, mBio 7:e02007-15, 2016) contributes to the understanding of the dynamic modulation of chromatin assembled on the herpes simplex virus genome by monitoring the levels of characteristic heterochromatic histone modifications (histone H3 lysine 9 and 27 methylation) associated with a model viral early gene during the progression of lytic infection. Additionally, this study builds upon previous observations that the viral immediate-early protein ICP0 plays a role in reducing the levels of heterochromatin associated with the early genes.

## COMMENTARY

In recent years, a plethora of cellular proteins involved in chromatin modulation have been identified. An impressive array of enzymes and specificity factors install and remove complex histone posttranslational modifications that lay the foundation for recognition by families of effector proteins (“histone readers”). Combined with histone chaperones, nucleosome remodelers, and chromatin domain organizers, these components ultimately integrate signaling and regulatory events that define gene expression patterns, control DNA replication and repair, and establish or maintain cell identity.

Like their host cells, many nuclear DNA viruses, including herpesviruses, are subject to the regulatory overlay of chromatin assembled on their genomes. These viruses counter, modulate, and utilize host cell chromatin machinery to regulate the coordinated expression of their genes in a program that supports a productive infection or establishes and maintains a latent-persistent state.

For some herpesviruses, studies of the global “epigenetic landscape” have revealed a complex arrangement of chromatin domains or subdomains within the genomes that exhibit heterochromatic characteristics, bivalent marks (repressive and activating histone signatures), or euchromatic states that correlate with repression or activation of the appropriate lytic or latency gene products. Importantly, transitions between these chromatin states are a component of the transitions between productive infection and persistence.

The characteristics and the mechanisms involved in the establishment and modulation of herpesviral chromatin are of intense interest, as (i) chromatin modulation is a critical regulatory parameter governing the infectious or persistent cycle of these viruses; (ii) the viral genomes are a microcosm of the cell genome, and important insights into chromatin modulation can be discerned from the study of these pathogens; (iii) these viruses encode proteins that interact with, activate, degrade, or alter the specificity of cellular chromatin machinery in unique manners; and (iv) targeting the components involved in modulation of viral chromatin could potentially provide novel approaches to control viral infection, latency, and reactivation.

Strikingly, herpesviruses are packaged in capsids in the absence of histone proteins. Thus, they enter the cell devoid of nucleosomes but rapidly undergo canonical nucleosome assembly (1). For herpes simplex virus (HSV), the initial chromatin state of the viral genome exhibits characteristics of heterochromatin (histone H3 lysine 9 [H3-K9] and H3-27 methylation) (2–4) and appears to be a response of the cell to the infecting nonnucleosomal genome.

This state is highly dynamic, and studies have implicated cell and/or viral factors, combined with histone modification and chromatin regulatory components, in modulating this dynamic. The balance of these competing regulatory components can determine the initiation of infection by either enhancing the epigenetic suppression of the viral genome or promoting a transition of this suppressive chromatin state to a permissive euchromatic one at the viral immediate-early (IE) genes to initiate this first wave of viral gene expression (5, 6).

In contrast, little is known concerning the chromatin state associated with the later classes (early, E; late, L) of viral genes and how chromatin is modulated to enable appropriate and timely transcription. Lee et al. (7) asked two questions concerning the observed patterning of chromatin associated with HSV E genes. The first addressed the kinetic assembly and removal of characteristic heterochromatin marks, defined by histone H3-K9 and H3-K27 methylation, on an HSV E gene (ICP8/UL29) promoter throughout the course of infection. The second investigated the impacts of viral IE regulatory protein ICP0 on the reduction in absolute levels of heterochromatic histone marks associated with these genes during induction of E gene expression.

Using chromatin immunoprecipitation (ChIP) assays to monitor total histone H3 levels and H3-K9 and H3-K27 methylation at time points from 1 to 12 h postinfection, the authors made several observations. First, while the absolute proportions of these repressive marks do not significantly change during the early stage of infection (1 to 4 h postinfection [hpi]), the total levels of nucleosomes associated with the viral E gene promoters decreases coincident with the induction of E gene expression (3 to 4 hpi). Thus, the expression of E genes may be dependent upon targeted and timely displacement of nucleosomes associated with E gene promoters. While it was not investigated here, it would be anticipated that multiple chromatin modulation factors (histone demethylases, acetyltransferases, histone chaperones, and remodelers) would play important roles in reducing the E gene nucleosomal load to assemble RNA polymerase II complexes and drive E gene expression. The mechanisms by which these factors would be recruited to E gene promoters has not been studied; however, this may be dependent upon multiple E gene activators, including the HSV major transcriptional activator ICP4.

A second observation is that the bulk of repressive H3-K9 and H3-K27 methylation associated with the E gene promoter is most significantly reduced via histone exchange during viral DNA replication, as the levels of both of these marks relative to the total histone H3 level decrease significantly at the onset of replication. Interestingly, the levels of histone H3 and H3-K9 methylation decrease when viral DNA replication is inhibited, while specific reduction in the levels of H3-K27 methylation appeared to be more highly dependent upon viral genome replication. The result is consistent with some observations that H3-K27 methylation has a relatively low turnover rate in the presence of inhibitors of the H3-K27 methyltransferase EZH2 (8, 9).

The results presented by Lee et al., however, raise a number of questions as to the significance or relevance of this modification in the E gene promoter region assessed in that study. Previously, work from the Knipe laboratory suggested that the histone H3-K27 demethylase JMJD3/KDM6B contributed to viral E gene expression (10). Therefore, it remains possible that (i) the specific positioning of H3-K27 methylation relative to viral gene coding regions or specific targeted changes were not detected in these ChIP assays or (ii) cycles of methylation-demethylation may occur that would be more readily revealed by ChIP assays under conditions under which the relevant methyltransferases or demethylases are depleted or inhibited. Alternatively, H3-K27 methylation may not be a critical repressive signal for E genes but could be a regulatory component of the DNA replication-dependent L genes. A determination of the relevance of these and other histone modifications for expression of the different classes of viral genes may depend on global mapping under conditions in which specific modification enzymes are depleted or inhibited.

A second component of this study relates to the impact of the virus-encoded transcriptional activator ICP0 on the levels of these two repressive marks. Several herpesviruses encode proteins that interact with, recruit, alter the specificity of, or degrade chromatin modulation components either to efficiently effect viral lytic gene expression or to promote the establishment of latency. HSV ICP0 is an IE protein that regulates multiple stages of the viral infectious cycle and is important for the promotion of viral gene expression. Several studies have suggested that one way in which this is accomplished is via directly or indirectly impacting chromatin associated with the viral genome. For example, (i) ICP0 antagonizes cellular antiviral responses, including promyelocytic leukemia-associated factors and interferon gamma-inducible protein 16, that may play a role in the assembly of repressive heterochromatin on the infecting viral genome (11–15), and (ii) ICP0 disrupts the repressive CoREST-histone deacetylase complex (16, 17). Together with the observation that overexpression of the CLOCK histone acetyltransferase can compensate for loss of ICP0 expression (18), these studies suggest that the activities of ICP0 result in enhanced levels of histone acetylation that would promote nucleosome remodeling or fluidity and, ultimately, gene expression.

**FIG 1:**
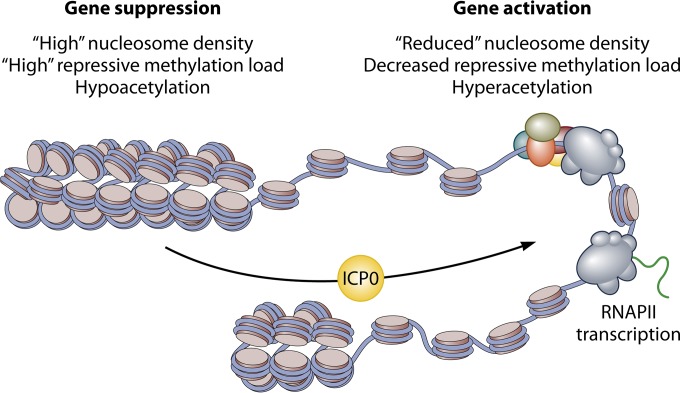
Infection of cells by HSV results in the accumulation of repressive heterochromatin on the viral genome. Lee et al. demonstrated that the HSV activator ICP0 is important for reducing the density of nucleosomes and thus the absolute levels of repressive histone methylation associated with viral E genes to promote E gene expression. RNAPII, RNA polymerase II.

Using two ICP0 mutant viruses, Lee et al. make a case that this regulator is necessary for the efficient early-stage reduction in the total levels of nucleosomes associated with an E promoter (Fig. 1). While the replication-dependent decreases in H3-K9 and H3-K27 methylation levels are also impacted, this is likely a consequence of the defect in DNA replication of these viruses. Thus, the study builds upon previous observations that one important function of ICP0 is to affect the acetylation levels and density of nucleosomes stably associated with the HSV genome (19). It will be interesting to determine whether the modulation of histone levels and repressive chromatin marks is direct, indirect, a function of the known activities of the protein, or mechanistically distinct from the previously described activities.
